# Global Molecular Analyses of Methane Metabolism in Methanotrophic Alphaproteobacterium, *Methylosinus trichosporium* OB3b. Part II. Metabolomics and 13C-Labeling Study

**DOI:** 10.3389/fmicb.2013.00070

**Published:** 2013-04-03

**Authors:** Song Yang, Janet B. Matsen, Michael Konopka, Abigail Green-Saxena, Justin Clubb, Martin Sadilek, Victoria J. Orphan, David Beck, Marina G. Kalyuzhnaya

**Affiliations:** ^1^Department of Chemical Engineering, University of WashingtonSeattle, WA, USA; ^2^Division of Biology, California Institute of TechnologyPasadena, CA, USA; ^3^Department of Chemistry, University of WashingtonSeattle, WA, USA; ^4^Division of Geological and Planetary Sciences; California Institute of TechnologyPasadena, CA, USA; ^5^eScience Institute, University of WashingtonSeattle, WA, USA; ^6^Department of Microbiology, University of WashingtonSeattle, WA, USA

**Keywords:** methanotrophic proteobacteria, serine cycle, ethylmalonyl-CoA pathway in methanotrophs, metabolic fluxes labeling

## Abstract

In this work we use metabolomics and ^13^C-labeling data to refine central metabolic pathways for methane utilization in *Methylosinus trichosporium* OB3b, a model alphaproteobacterial methanotrophic bacterium. We demonstrate here that similar to non-methane utilizing methylotrophic alphaproteobacteria the core metabolism of the microbe is represented by several tightly connected metabolic cycles, such as the serine pathway, the ethylmalonyl-CoA (EMC) pathway, and the citric acid (TCA) cycle. Both *in silico* estimations and stable isotope labeling experiments combined with single cell (NanoSIMS) and bulk biomass analyses indicate that a significantly larger portion of the cell carbon (over 60%) is derived from CO_2_ in this methanotroph. Our^13^ C-labeling studies revealed an unusual topology of the assimilatory network in which phosph(enol) pyruvate/pyruvate interconversions are key metabolic switches. A set of additional pathways for carbon fixation are identified and discussed.

## Introduction

Microbial oxidation of methane is one of the key elements of the global carbon cycle. The ability to oxidize methane has been demonstrated in two classes of Proteobacteria, Alpha, and Gamma. *Methylosinus trichosporium* OB3b was first described by Whittenbury et al. (1970) and has served as a model system for the investigation of methane utilization in obligate alphaproteobacterial methanotrophs for decades (Lawrence and Quayle, 1970; Strom et al., 1974; Cornish et al., 1984; Jollie and Lipscomb, 1991; Park et al., 1991, 1992; DiSpirito et al., 1998; Lontoh and Semrau, 1998; Gilbert et al., 2000; Trotsenko and Murrell, 2008). While the key metabolic pathways for carbon assimilation in *M. trichosporium* OB3b have been predicted (Strom et al., 1974), several fundamental questions have never been answered, such as how cells regenerate glyoxylate (Anthony, 1982), what is the role of the TCA cycle in methanotrophic metabolism is (Patel et al., 1979; Shishkina and Trotsenko, 1982), and why CO_2_ supplementation has a significant positive effect on cell growth (Park et al., 1991, 1992). A draft genome of *M. trichosporium* OB3b has recently been generated (Stein et al., 2010), providing a genetic framework for characterization of the methanotrophy. In this work, models of carbon assimilation pathways in *M. trichosporium* OB3b predicted by biochemical characterization (Strom et al., 1974; Trotsenko and Murrell, 2008) and global gene expression analysis (Matsen et al., 2013) are further tested by metabolomic and ^13^C-labeled studies.

## Results and Discussion

### Growth parameters and incorporation from methane and CO_2_ via ^13^C-labeling experiments

A strong dependence of the *Methylosinus trichosporium* OB3b growth upon CO_2_ supplementation has been previously demonstrated (Park et al., 1991, 1992). Similarly, a lag-period (up to 24 h) was observed in methane supplemented cultures inoculated at low cell density (OD_600_ < 0.05) at ambient concentrations of CO_2_. The initial lag-period was shortened by the addition of CO_2_ (5% of head space). Furthermore, the specific growth rate was also increased to 0.057 ± 0.002 h^−1^ (versus μ = 0.038 ± 0.004 h^−1^, in cultures grown without additional CO_2_). CO_2_ supplementation did not have any significant effect on the final cell density (data not shown).

It has been speculated that the positive effect of CO_2_ is a result of the high demand of the serine pathway for CO_2_ (Park et al., 1991, 1992). However, addition of extra CO_2_ does not affect the growth of non-methane utilizing methylotrophs such as *Methylobacterium extorquens* AM1 with the serine pathway for carbon utilization (Yanfen Fu, Mary E. Lidstrom personal communication). Several putative carboxylating systems were identified in the *M. trichosporium* OB3b genome: phosphoenolpyruvate carboxylase (Ppc); two propionyl-CoA carboxylases; crotonyl-CoA reductase (*ccr*); NAD(P)-dependent malic enzyme (*mae*), acetyl-CoA carboxylase (*accABD*), phosphoribosylaminoimidazole carboxylase, pyruvate carboxylase (*pcx*), and a putative 2-oxoacid ferredoxin oxidoreductase. To investigate the fate of CO_2_ in OB3b, we estimated the C_1_-carbon incorporation derived from methane (at the level of methylene-tetrahydrofolate, MeH4F) and/or bicarbonate into biomass using stable isotope labeling experiments and bulk biomass analyses. For these experiments, cells of *M. trichosporium* OB3b were grown on ^13^CH_4_ (20%) with or without external CO_2_ supplementation (5% atmosphere). A Cameca nanoSIMS 50 L was used to analyze the ^13^C/^12^C ratio of individual cells from either methane only or methane + carbon dioxide treatment. Individual *M. trichosporium* OB3b cells from the ^13^CH_4_ treatment showed substantial ^13^C enrichment (^13^C/^12^C ratio of 0.351 ± 0.231), albeit with significant cell-to-cell variation (Table 1). Cells grown on ^13^CH_4_ in the presence of added ^12^CO_2_ showed less cell-to-cell variation. The amount of ^13^C-enriched biomass derived from methane dropped dramatically in the presence of elevated CO_2_ (^13^C/^12^C ratio of 0.134 ± 0.046), reflecting the reincorporation of ^13^CO_2_ derived from ^13^CH_4_ when ^12^CO_2_ is not elevated (Crowther et al., 2008; Table 1). Analysis of bulk cell pellets from *M. trichosporium* OB3b cultures incubated under the same conditions (^13^CH_4_ with and without elevated ^12^CO_2_) also showed a similar trend with significantly lower ^13^C enrichment in biomass from cultures incubated with additional CO_2_ relative to treatments with only methane (data not shown). Overall, ^13^C-labeling data suggest significant assimilation of CO_2_ relative to CH_4_. Assuming that in the presence of 5% ^12^CO_2,_ no reincorporation of ^13^CH_4_-derived ^13^CO_2_ occurred, our results suggest that at least 62% of the assimilated carbon is from CO_2,_ significantly greater than the 50% observed for *M. extorquens* AM1, a non-methane utilizing serine cycle methylotroph (Peyraud et al., 2011).

**Table 1:** **^13^Carbon enrichment of *Methylosinus trichosporium* OB3b cells grown on ^13^C-labeled methane with or without additional supplementation with unlabeled carbon dioxide**.

Treatment	^13^C/^12^C ratio	Mean	Lower 95%	Upper 95%	Number of cells analyzed
^13^C-methane	0.351 ± 0.231	0.04	0.264	0.437	30
^13^C-methane + ^12^CO_2_ (5%)	0.134 ± 0.046	0.01	0.113	0.155	21

*^13^C of cells grown on unlabeled ^12^CH_4_ is 55.19 ± 0.21*.

### Assimilation: *in silico* and metabolomics studies

**Figure 1:**
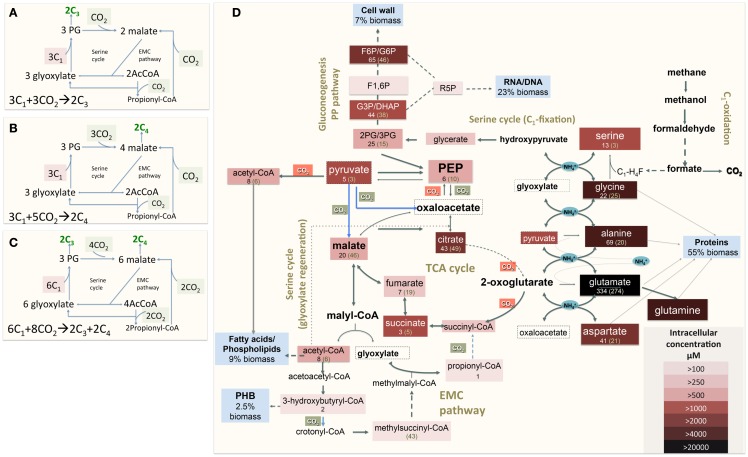
**(A)** Schematic representation of the serine cycle with the EMC pathway for carbon assimilation [adapted from (Peyraud et al., 2009, 2011)]. **(B)** Schematic representation of the serine cycle with the EMC pathway for carbon assimilation (adapted from Anthony, 2011); **(C)** Schematic representation of the serine cycle with the EMC pathway for carbon assimilation in *Methylosinus trichosporium* OB3b (see description in the text). **(D)** Central metabolism of *Methylosinus trichosporium* OB3b grown on methane as deduced from the genome sequences, transcriptomic, and metabolomic studies. Metabolites boxed were measured. Box color is an indicator of the metabolite pool, with darker colors indicating a larger pool. Numbers under a metabolite names are rates of ^13^C-carbon incorporation: the first number is the rate of CH_4_-derived carbon; number in parentheses- is the rate of CO_2_-derived carbon incorporation. PHB-polyhydroxybutyrate; F1, 6P, Fructose-1, 6-bisphosphate; F6P/G6P, fructose-6-phosphate/glucose-6-phosphate; GAP/DHAP, glyceraldehyde-3-phosphate/dihydroxyacetone phosphate; 2PG/3PG, 2-phosphoglycerate/3-phosphoglycerate.

The functional organization of the *M. trichosporium* OB3b metabolic network operating during growth on methane has been discussed in an accompanying manuscript by Matsen et al. (2013). A summary of the central metabolism is shown in Figure 1. The chemical composition of *M. trichosporium* OB3b (RNA, DNA, protein, polyhydroxybutyrate (PHB), fatty acids, intracellular metabolites) was determined experimentally or taken from available literature (Weaver et al., 1975; Williams, 1988; Guckert et al., 1991; Sun and Wood, 1996; Lloyd et al., 1999; Doronina et al., 2008). Proteins comprise a significant portion of the cell dry weight (55%). The specific amino acid composition of the cell proteins was estimated from RNA-seq data, and the data were used to estimate contribution of C2-C6 intermediates into biomass (shown in Figure 1, Table A1 in Appendix).

As background information for physiological pathways, 27 targeted intermediates involved in the serine cycle, EMC pathway, TCA cycle, and gluconeogenesis were quantified (Table 2). Among those, the lowest concentration was observed for propionyl-CoA (108.6 μM) and the highest concentration was 24 mM for glutamate (Table 2). Relatively high concentrations of pyruvate (1 mM) and its corresponding amino acid, alanine (4.5 mM) were detected.

**Table 2:** **Intracellular pool of key metabolites in *M. trichosporium* OB3b**.

Metabolite	Concentration μM	SD
**SERINE CYCLE AND ETHYLMALONYL-CoA PATHWAY**		
Serine	1504.9	142.0
Glycine	4631.9	484.1
Glycerate	365.4	41.8
Phosphoglycerate	811.9	118.3
3-Hydroxybutyryl-CoA	185.2	54.3
Methylsuccinic acid	119.7	37.6
Phosphoenolpyruvate	661.5	103.4
Propionyl-CoA	108.6	26.5
**TCA CYCLE AND RELATED AMINO ACIDS**		
Acetyl-CoA	519.3	162.9
Citrate	3149.2	528.1
Fumarate	400.5	85.7
Malate	950.2	154.7
Succinate	1304.4	323.9
Succinyl-CoA	320.0	80.7
Aspartate	1998.0	481.7
Glutamate	24034.1	4060.4
Glutamine	9864.0	2457.3
**GLUCONEOGENESIS/PYRUVATE-PEP AND RELATED AMINO ACIDS**		
Fructose-1,6-bisphosphate	136.4	37.6
Fructose-6-phosphate/Glucose-6-phosphate	2836.0	654.3
Glyceraldehyde-3-phosphate/Dihydroxyacetonephosphate	1968.6	509.6
Phosphoenolpyruvate	661.5	103.4
Pyruvate	1134.7	299.3
Ribulose-5-phosphate/Ribose-5-phosphate	140.6	57.1
Alanine	4495.5	766.2

### Metabolome analysis: ^13^C-labeling

**Figure 2:**
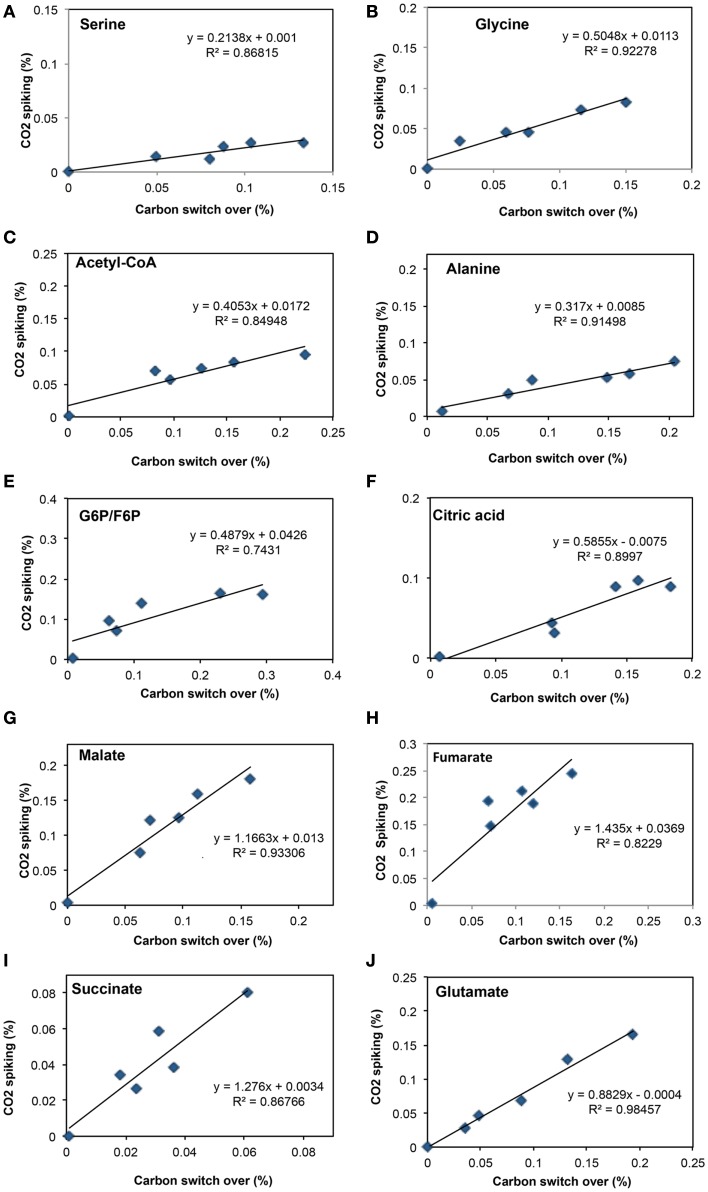
**Comparison of total ^13^C-incorporation (%) between switch from ^12^CH_4_ to ^13^CH_4_ versus that of ^13^CO_2_ spiking in a time course (0, 1, 2, 5, 10, 20 min)**. In each panel, the total 13C-incorporation under carbon switchover and CO_2_ spiking conditions are plotted against each other for each time point. The six points corresponding to 0, 1, 2, 4, 10, and 20 min were best fit and the slope of the resulting line used to estimate the relative rate of incorporation into serine **(A)**, glycine **(B)**, acetyl-CoA **(C)**, alanine **(D)**, glucose-6-phosphate/fructose-6-phosphate **(E)**, citric acid **(F)**, malate **(G)**, fumarate **(H)**, succinate **(I)**, and glutamate **(J)**.

**Figure 3:**
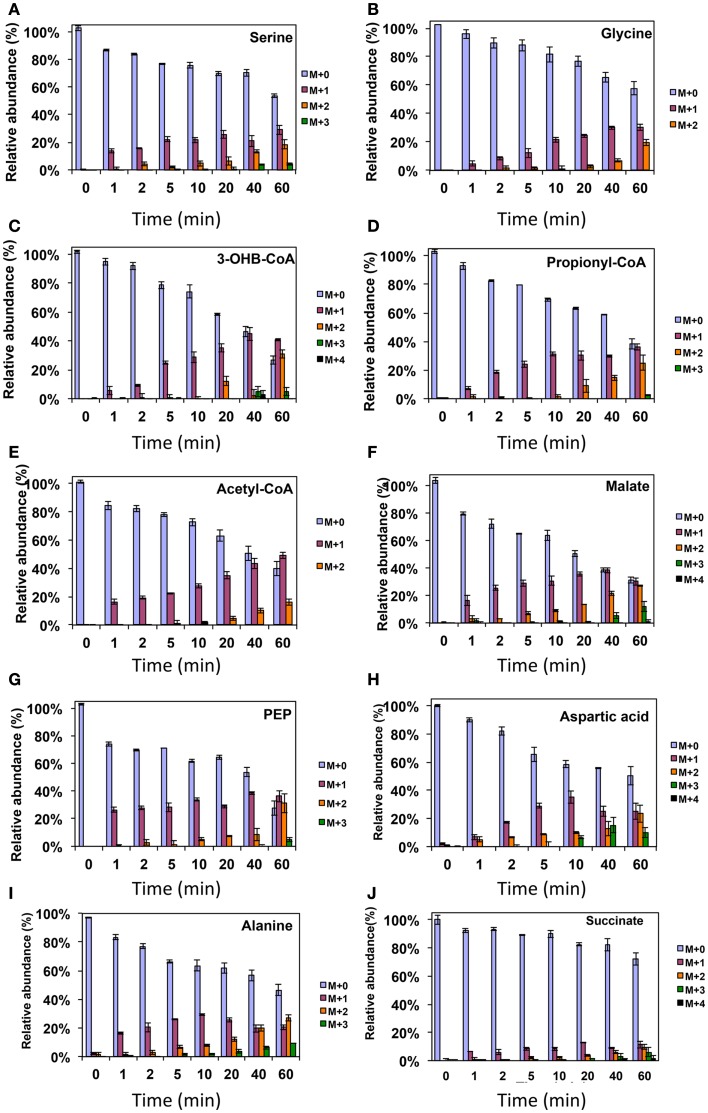
**^13^C-incorporation during the switch from ^12^CH_4_ to ^13^CH_4_ in *Methylosinus trichosporium* OB3b**. **(A)** ^12^C/^13^C-isotopomer distributions of serine **(A)**, glycine **(B)**, 3-hydroxybutyric acid **(C)**, propionyl-CoA **(D)**, acetyl-CoA **(E)**, malate **(F)**, phosphoenolpyruvate **(G)**, aspartic acid **(H)**, alanine **(I)**, and succinate **(J)**. Additional data are shown in Figure A1 in Appendix. M + 0 represented non-labeled compound, M + 1 represented compound with one ^13^C-label, M + 2 represented compound with two ^13^C-labels and so on.

**Figure 4:**
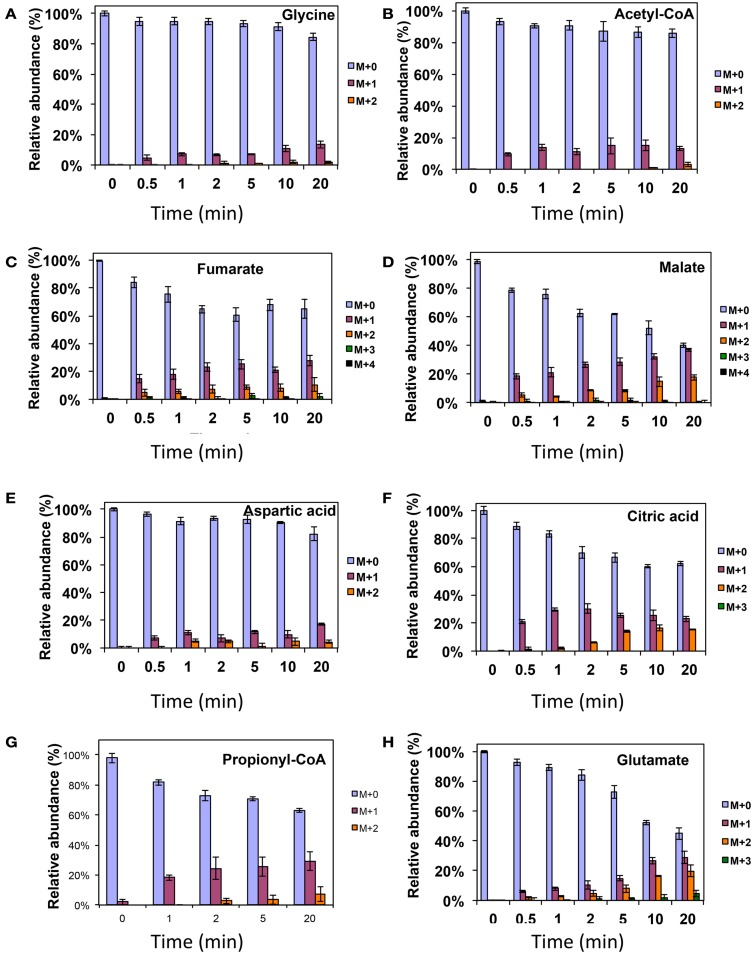
**^13^C-incorporation during ^12^CO_2_ spiking in *Methylosinus trichosporium* OB3b grown on ^12^CH_4_**. ^12^C/^13^C-isotopomer distributions of glycine **(A)**, acetyl-CoA **(B)**, fumarate **(C)**, malate **(D)**, aspartic acid **(E)**, citric acid **(F)**, propionyl-CoA **(G)**, glutamate **(H)**. Additional data are shown in Figure A2 in Appendix. *M* + 0 represented non-labeled compound, *M* + 1 represented compound with one ^13^C-label, *M* + 2 represented compound with two ^13^C-labels and so on.

**Figure A1:**
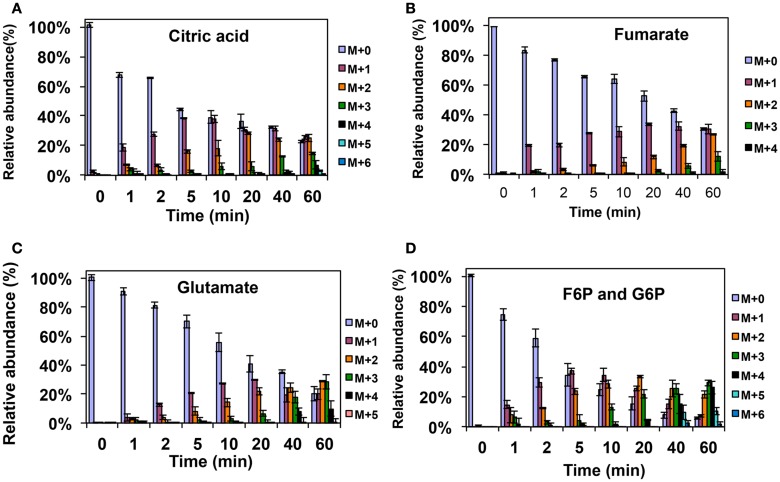
**^13^C-incorporation during the switch from ^12^CH_4_ to ^13^CH_4_ in *Methylosinus trichosporium* OB3b**. **(A)** ^12^C/^13^C-isotopomer distributions of citric acid **(A)**, fumarate **(B)**, glutamate **(C)**, and fructose-6-phosphate/glucose-6-phosphate **(D)**.

To address some of the hypotheses generated by gene expression and *in silico* studies and to probe metabolic pathways for methane and CO_2_ assimilation in *M. trichosporium* OB3b, we monitored the dynamic ^13^C-incorporation of intermediates through two different ^13^C-tracing experiments: switching from ^12^CH_4_ to ^13^CH_4_ (*switch* experiment) and spiking ^13^CO_2_ into cell cultures grown on ^12^CH_4_ (*spike* experiment) (Figures 2–4, Figure A1 in Appendix). The data generated were compared with predicted pathways for carbon assimilation (summarized in Figure 1, see also Matsen et al., 2013). All key intermediates of central pathways could be divided into three groups: (1) efficiently labeled with methane-derived carbon, such as serine, alanine, G6P/F6P, citric acid (Figures 2A–F); (2) more efficiently labeled with CO_2_-derived carbon, such as malate, fumarate, and succinate (Figures 2G–I); and (3) similarly labeled by both CO_2_ and CH_4_ carbon, such as glutamate (Figure 2J).

As was expected during the switch from ^12^CH_4_ to ^13^CH_4_, singly labeled serine was generated quickly, demonstrating that any unlabeled glycine rapidly reacted with labeled methylene H_4_F (Figure 3). Doubly labeled serine appeared later, followed by triply labeled serine. About 25% of the total serine pool (as determined by normalization to total pool of serine) was labeled after 20 min. The low ^13^C-incorporation suggested a slow uptake that was consistent with the methane consumption rate (in *M. trichosporium* OB3b). The labeling distribution of glycine (herein an indicator of glyoxylate) is shown in Figures 3B and 4A. Around 25% of the glycine (an indicator of glyoxylate) was labeled in 20 min in the switch experiment. Ten percent of the singly label glycine occurred after 10 min in the ^13^CO_2_ spiking experiments (Figure 4A), which is also consistent with regeneration of glyoxylate via the EMC pathway (Figure 4).

In addition, two metabolites involved in the EMC pathway [3-OHB-CoA (entry) and propionyl-CoA (exit)] were monitored during CH_4_ spike (Figures 3C,D). Both compounds were singly labeled quickly. These could be generated from one singly labeled acetyl-CoA and one unlabeled acetyl-CoA (Figure 3E). The low incorporation for the second carbon can be explained by the additional synthesis of unlabeled acetyl-CoA via the EMC pathway itself. ^13^CO_2_-carbon was also quickly incorporated into propionyl-CoA (Figure 4G). These data also provide metabolic proof of EMC operation in the strain.

About 40% of the total malate pool was labeled throughout the course of the ^13^CH_4_-experiments (Figure 3F). Both methane and CO_2_ (generated from ^13^CH_4_-oxidation) can contribute to the label. However, the rate of CO_2_ incorporation into malate and fumarate was high too (Figures 2G,H). The possible routes for the CO_2_ incorporation into malate labeling are through the serine cycle, EMC pathway and carboxylation of pyruvate (Figure 1). As shown in Figure 4D, singly labeled malate increased to around 20% after 0.5 min and remained at a similarly high percentage throughout the experiment, adding support to the hypothesis that the carboxylation via the EMC pathway and pyruvate contributes significantly to the pool of malate in addition to PEP-carboxylation. The labeling pattern of fumarate favors the hypothesis of the EMC pathway is a major source of malate. However, it should be noted that the ^13^CO_2_-fumarate labeling may be explained by the reversible reaction catalyzed by *fumC*. On the other hand, the rate of CO_2_ incorporation into aspartate (herein an indicator of oxaloacetate) was slow compared to malate (Figures 4D,E). The rates of C_1_-incorporation (^13^CH_4_-derived carbon) into malate and aspartate are almost identical in the switch over experiment (Figures 3F,H). Thus, the much slower rate of CO_2_ incorporation into aspartate at the early time points could not be attributed only to the large pool of the compound. The labeling patterns of malate and aspartate during ^13^CO_2_-spikes indicate that at least one of the compounds (most likely malate) comes from alternative sources. Doubly labeled malate was observed throughout the experiment (Figure 4D). The reversible conversions of malyl-CoA to malate and malate to fumarate may result in fumarate scrambling and contribute to doubly labeled malate. However, ^13^CO_2_ is incorporated into fumarate and succinate more efficiently than C_1_-labeled carbon (Figures 2H,I). Together the presented data indicate that the ECM pathway may serve as a significant source of intracellular malate.

**Figure A2:**
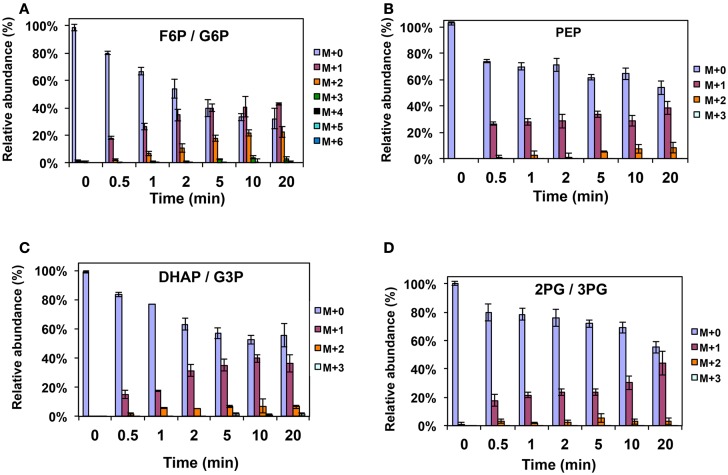
**^13^C-incorporation during ^12^CO_2_ spiking in *Methylosinus trichosporium* OB3b grown on ^12^CH_4_**. ^12^C/^13^C-isotopomer distributions of fructose-6-phosphate/glucose-6-phosphate **(A)**, phosphoenolpyruvate **(B)**, dihydroxyacetone phosphate/glyceraldehyde 3-phosphate **(C)**, 2-phosphoglycerate/3-phosphoglycerate **(D)**.

According to current pathway prediction, both acetyl-CoA and glyoxylate are generated from malyl-CoA. The labeling rate of these two compounds was similar during the switch over experiments (Figures 3B,E). The incorporation of CO_2_-labeling into intracellular acetyl-CoA was slow compared to the ^13^C-carbon label that originated from methane. The labeling pattern of acetyl-CoA is similar to other intermediates of the serine cycle generated upstream from phosphoenolpyruvate, including all intermediates of gluconeogenesis/PPP, and pyruvate/alanine (Figures 3C,I; Figures A1D and A2A-D in Appendix). As a rule, these intermediates were labeled with methane-derived carbon faster than with CO_2_ (Figure 2). This suggests that significant flux of carbon assimilated as MeH_4_F is incorporated into biomass via gluconeogenesis/PPP and that pyruvate contributes to at least 1/3 of the intracellular pool of acetyl-CoA. Such conversion seems to be essential in the strain, which is known to have high demand for acetyl-CoA as a key metabolite for membrane and PHB biosynthesis.

Singly and doubly ^13^C_1_-labeled citric acid and glutamate were generated quickly, followed by triply and quadruple labeled variants (Figures 3 and 4). About 32% of the total pool of citric acid was labeled throughout the experiments demonstrating significant flux through the forward TCA cycle. During ^13^CO_2_ experiments, singly labeled citric acid appeared quickly at the early time points most likely as a result of CO_2_ incorporation into oxaloacetate (Figure 4F). The concentration of doubly labeled citric acid eventually increased to 15%, yet triply labeled citric acid (the limit of detection of citric acid was less than 0.2 pmol on column) was not clearly detected within 20 min. For glutamate, singly and doubly labeled patterns rose slowly due to its high concentration (Figure 4H). However, 4.7% of the glutamate pool (567 ± 268 μM) was triply labeled after 20 min. ^13^C-labeling data indicate that cells could produce α-ketoglutarate from succinyl-CoA. The genome analysis suggests one system that may perform this function, namely related to 2-oxoacid ferredoxin oxidoreductase (METTOv1_1080004). This gene is expressed (Matsen et al., 2013). The presence of a partially reversible TCA has never been predicted for methanotrophic bacteria and represents an interesting subject for follow-up studies.

## Conclusion

In this work we used ^13^C-labeling to test and refine the previous genome/transriptome based reconstruction of the central metabolic pathways in *Methylosinus trichosporium* OB3b grown on methane (Matsen et al., 2013). While some metabolic functions correlate well with previous enzymatic and genetic studies, several novel functions are newly predicted. The major outcomes of our work are listed below:

1. *M. trichosporium* OB3b uses the EMC variant of the serine cycle for carbon assimilation. Both *in silico* and *in vivo* labeling data indicate that a significant fraction of biomass comes from C_4_-metabolites (after carboxylation step). Thus the overall balance of the pathway is shifted to a higher CO_2_-assimilation mode, whereby eight molecules of CO_2_ are consumed per six molecules of MeH4F (Figure 1C). The data also suggest that a significant fraction of methane oxidized to CO_2_ is incorporated back via the EMC pathway. Thus this methanotroph differs from facultative methanol utilizers with respect to overall CO_2_ assimilation. Type II methanotrophs may represent a better system for C_1_-based commercial production of chemicals than serine cycle methylotrophs, as the methanotrophs will reincorporate more CO_2_ per unit of substrate oxidized.
2. Both genomic and trancriptomic data predict a variety of reactions at the PEP-pyruvate-oxaloacetate node. Metabolomic data indicate that the metabolic interconversions play an important role in the distribution of carbon flux between the major metabolic pathways. A significant fraction of PEP is converted to pyruvate, which serves as a precursor for alanine and is also used as an acceptor in two anaplerotic CO_2_-fixation reactions: pyruvate carboxytransferase and malic enzyme. ^13^C-labeling data strongly suggest that a part of the intracellular pool of the acetyl-CoA comes from pyruvate most likely via pyruvate dehydrogenase. The contribution of pyruvate to acetyl-CoA has never been discussed before, as it was assumed that the serine cycle refills the cellular needs for this intermediate.
3. A significant fraction of intracellular malate comes from the EMC pathway. The data suggest that the serine cycle is split into two functional branches that have different metabolic control. The first, labeled as “C1-fixation” part (Figure 1), includes all steps from glycine to PEP. This branch of the assimilatory pathway contributes to 53% of biomass. Most of the C1-carbon incorporated at first step of the branch is directed to gluconeogenesis (RNA/DNA, cell wall biosynthesis) and amino acid (serine, cysteine, tryptophan tyrosine, and phenylalanine) biosynthesis. The second part of the serine cycle (named “glyoxylate regeneration” in Figure 1) overlaps with the EMC pathway. Our data suggest the EMC pathway contributes significantly to replenish the intermediates (mostly malate, acetyl-CoA, and glyoxylate) of this part of the assimilation.

## Experimental Procedures

### Strain and cultivation conditions

*Methylosinus trichosporium* strain OB3b was kindly provided by Dr. Lisa Stein. The culture was grown in 250 mL glass bottles on modified NMS that contained the following contents (Whittenbury et al., 1970): 1 g KNO_3_, 1 g MgSO_4_⋅7H_2_O, 0.134 g CaCl_2_⋅2H_2_O, 0.25 g KH_2_PO_4_, 0.7 g Na_2_HPO_4_⋅12H_2_O, and 2 mL of trace elements solution. The trace elements solution contained 0.5 g Na_2_-EDTA, 1.0 g FeSO_4_⋅7H_2_O, 0.75 g Fe-EDTA, 0.8 g ZnSO_4_⋅7H_2_O, 0.005 g MnCl_2_⋅4H_2_O, 0.03 g H_3_BO_3_, 0.05 g CoCl_2_⋅6H_2_O, 0.4 g Cu-EDTA, 0.6 g CuCl_2_⋅2H_2_O, 0.002 g NiCl_2_⋅6H_2_O, and 0.05 g Na_2_MoO_4_⋅2H_2_O in 1 L of water. The bottles were sealed with rubber stoppers and aluminum caps. 50 mL of methane were added to the 200 mL headspace. If necessary, 10 mL of CO_2_ were added to the headspace. Bottles were shaken at 250 RPM at 30°C for 1–4 days.

### Growth parameters and methane consumption rate measurements

Methane consumption rates and cell density (OD_600_) were measured in triplicate as cultures grew. Methane measurements were collected on a Shimadzu Gas Chromatograph GC-14A, using FID detection with helium as the carrier gas. Concentrations were deduced from standard curves. OD_600_ was measured on a Beckman DU^®^ 640B spectrophotometer in plastic 1.5 mL cuvettes with a 1 cm path length.

### ^13^CO_2_ and ^13^CH_4_ labeling experiments for single cells and bulk biomass: EA-IRMS and NanoSIMS analyses

Experiments to determine the relative contributions of methylene H_4_F (produced from methane) and CO_2_ to labeled biomass were performed by modifying a previous method (Crowther et al., 2008). Cells were grown in NMS medium supplemented with either 20% ^13^CH_4_, 20% ^12^CH_4_, 20% ^13^CH_4_ and 5% ^12^CO_2_, or 20% ^12^CH_4_ and 5% ^13^CO_2_. After initial incubations at the described gas mixtures, cell cultures (OD_600_ = 0.4) were diluted (1:50) with fresh medium and transferred into new vials containing the same gases. For bulk and single cell NanoSIMS measurements, cells were harvested at OD_600_ = 0.4.

### Bulk δ^13^C isotopic analysis via elemental analysis coupled to isotopic ratio mass spectrometry

For bulk elemental analysis coupled to isotopic ratio mass spectrometry (EA-IRMS) measurements, harvested cell pellets were initially frozen at −80°C, then lyophilized prior to δ^13^C analysis. As these were isotopically enriched samples, cell material was initially diluted in unlabeled glucose in order to lower the δ^13^C values to an acceptable range for the instrument. Specifically, 2 mg of dry cell mass was re-suspended in a glucose solution (1.455 M final concentration, Mallinckrodt Chemicals) and diluted to create a 3 point standard curve. Samples were then aliquoted into tin capsules, dried under desiccant, and sealed. The δ^13^C values of these samples were measured using an ECS 4010 Elemental Analyzer (Costech, Valencia, CA, USA) connected to a Finnigan ThermoQuest Delta^plus^XL IRMS and using CO_2_ as reference gas. δ^13^C values were calculated using the ISODAT 2.0 software (Thermo-Fisher Scientific, Bremen, Germany) and are reported in permil (‰) relative to the VPDB standard. Blank tin capsules along with urea and acetanilide standards of known δ^13^C value were analyzed after every 15 samples as well as at the beginning and end of each run in order to track instrument accuracy and precision.

As the cell cultures were diluted in unlabeled glucose, the raw δ^13^C values represented that of cellular carbon + glucose, and a back calculation was necessary in order to determine the δ^13^C of the cellular carbon. Three-point pure glucose standard curves were run in order to generate a linear regression of μmoles carbon versus peak area. The resulting equation was then used to determine the total μmoles carbon (glucose + cells) added to each sample run, in order to calculate μmoles of cellular carbon added. This value was then used to determine the δ^13^C of the cellular carbon.

### δ^13^C isotopic analysis via nanoscale secondary ion mass spectrometry

For bulk and single cell Nanoscale Secondary Ion Mass Spectrometry (NanoSIMS) measurements, cells were harvested at OD_600_ = 0.4 and fixed in 3% formaldehyde. Fixed cells were then washed in sterile buffer and deposited onto indium tin oxide (ITO) coated glass slides. Samples were analyzed using a CAMECA NanoSIMS 50 L housed at Caltech, using a mass resolving power approximately 5,000. A primary Cs^+^ ion beam (2.5 pA) was used to raster over target cells, and ion images ranging from 5 to 20 μm were collected at 256 × 256 pixel resolution with a dwell time of 14,000 ct/pixel. Several masses were collected in parallel including: ^12^C^−^, ^13^C^−^, ^14^N^12^C^−^, and ^14^N^13^C^−^ using electron multiplier detectors. Resulting ion images were processed using L’Image software (http://www.dtm.ciw.edu/users/nittler/limage_manual.pdf). In order to compare bulk (average δ^13^C over entire rastor) and single cell δ^13^C values, NanoSIMS targets included both aggregated cells as well as less densely populated areas of the slide containing single cells. δ^13^C values for single cells were calculated by creating “regions of interest” around individual cells in L’Image, using a 0.636 micron diameter circle which matched the ^12^C^14^N count space for an individual cell.

CO_2_ incorporation was determined by assuming that the ^13^C in the cells incubated with 5% ^12^CO_2_ was solely from direct ^13^CH_4_ incorporation, while in the sample without external ^12^CO_2_, incorporation of ^12^CO_2_ was minimal. Instead, CO_2_ incorporation in the latter samples was assumed to be from ^13^CO_2_ generated from ^13^CH_4_. These assumptions have been shown to be valid for the serine cycle methanol-utilizer, *M. extorquens* AM1 (Crowther et al., 2008). It was not possible to carry out experiments with *M. trichosporium* OB3b in the absence of any external CO_2_ to confirm these assumptions directly, since this strain grows poorly under these conditions. Therefore the calculation represents a minimum, and may be higher.

### *In silico* amino acid usage estimation

The amino acid usage data were generated from the codon usage as follows: nucleotide sequences for each predicted ORF were split into consecutive codons. The start and stop codons were ignored. The frequency of each codon was tabulated for each ORF. The amino acid usage table for each predicted protein product was multiplied by the mean number of reads mapped from replicates one and two. For each amino acid, the aggregate sum across predicted protein products was then normalized by the aggregate sum of all amino acids. Those fractions were also weighted by the neutral amino acid mass to predict the percent of the total pool by weight. The contribution of each amino acid into biomass was re-calculated using the estimation that proteins comprise 55% of biomass. The data are shown in Table A1 in Appendix.

### Metabolomics: Chemicals, sampling, and metabolite extraction

Acyl-CoAs, amino acids, organic acids, and sugar phosphates used as standards were of analytical grade and obtained from Sigma (St. Louis, MO, USA). Absolute ethanol (EMD Chemicals; Gibbstown, NJ, USA) was used in the metabolite extraction. All solvents used for liquid chromatography were LC-MS grade (Fisher Scientific; Fair Lawn, NJ, USA). Millipore purified water was used in the preparation of standard and sample solutions. For GC-MS sample derivatization, pyridine was purchased from EMD Chemicals (Gibbstown, NJ, USA), the trimethylsilylation (TMS) reagent [*N,O*–Bis (trimethylsilyl) trifluoroacetamide (BSTFA) + trimethylchlorosilane (TMCS), 99:1] and methoxyamine hydrochloride were both obtained from Sigma (St. Louis, MO, USA).

Samples (3 mL) of mid-exponential cultures (OD_600_ = 0.35 ± 0.02) were rapidly harvested by vacuum filtration using S-Pak™ membrane filters (0.22 μm, 47 mm) (Millipore; Billerica, MA, USA) and washed with 3 mL of fresh medium. The filter was immediately transferred to a petri dish located on the surface of a Cool Beans Chill Bucket™ (ISC Bioexpress; Kaysville, UT, USA) at −5°C. To collect cells, the following three sequential rinse solutions were applied: (i) 0.5 mL of 25 mM ice cold HEPES buffer (pH 5.2), (ii) 0.5 mL of −20°C ethanol solution (75/25, v/v, ethanol/aqueous 25 mM HEPES buffer, pH 5.2), and (iii) 1.5 mL of −20°C ethanol. The resulting solution was transferred to a precooled tube and stored in a −80°C freezer until it was ready for subsequent extractions.

Extraction of metabolites from *M. trichosporium* OB3b samples were carried out as previously published for *M. extorquens* AM1 (Yang et al., 2009). Briefly, the samples were incubated in a 100°C water bath for 3 min. The extracted cell suspension was cooled on ice for 5 min, then cell debris was removed by centrifugation at 5,000 RPM (4,300 × *g*) for 5 min. The cell-free metabolite extract was centrifuged at 14,000 RPM (20,800 × *g*) for 8 min. The supernatant was dried in a vacuum centrifuge (CentriVap^®^ Concentrator System; Labconco, MO, USA) and stored at −80°C. For LC-MS/MS analysis, each dried sample was dissolved in 50 μL of purified water. For GC-MS analysis, each sample was further derivatized in two steps. First, keto group were methoximated by adding 50 μL of methoxyamine solution (25 mg/ml methoxyamine hydrochloride in pyridine) and incubated at 60°C for 30 min. Second, trimethylsilylation was performed by adding 50 μl of a TMS reagent (BSTFA/TMCS, 99:1) and incubated at 30°C for 90 min.

### Metabolite measurement and absolute quantification

LC-MS/MS experiments were carried out on a Waters© (Milford, MA, USA) LC-MS system consisting of a 1,525 μ binary HPLC pump with a 2,777°C autosampler coupled to a Quattro *Micro*™ API triple-quadrupole mass spectrometer (Micromass^®^; Manchester, UK). The HILIC method employing gradient elution was carried out using the previously described column (Luna NH_2_, 250 mm × 2 mm, 5 μm; Phenomenex^®^; Torrance, CA, USA) and nearly identical conditions as described below. Gradient elution was carried out with 20 mM ammonium acetate +0.35% NH_4_OH (28%) in water (v/v)/acetonitrile (95:5, v/v) with pH 9.7 (mobile phase A), and acetonitrile (mobile phase B). The linear gradients used were 85–0% B for 15 min, 0% B for 11 min, 0–85% B for 1 min, and 85% B for 15 min. The total run time was 42 min at 0.15 mL/min. The injection volume was 10 μL. The eluent from each LC column was directed into the ion source of a mass spectrometer. The multiple reaction monitoring (MRM) experiments were carried out as previously described (Yang et al., 2010). The dwell time for each MRM transition was 0.08 s. All peaks were integrated using MassLynx™ Applications Manager (version 4.1) software.

GC-MS experiments were performed using an Agilent 7890/5975C GC-MS (Agilent Corp; Santa Clara, CA, USA). The column was HP-5MS (30 m × 0.25 mm × 0.25 μm film; Restek; Bellefonte, PA, USA). Ultra high purity helium was used as a carrier gas at a constant flow rate of 1 mL/min, 1 μL of a given sample was injected in split-less mode through an Agilent 7890 auto sampler. The inlet temperature was set to 250°C. The temperature began at 60°C, was held for 0.25 min, and then increased at 8°C/min to 280°C where it held for 10 min. The ion source temperature was set to 250°C. Mass spectra were collected from m/z 50–500 at 3 spectra/s with a 7.4 min solvent delay. The peaks were evaluated using Agilent data analysis software.

The absolute intracellular metabolite quantification was determined using an isotope ratio-based approach as previously published (Yang et al., 2010). Briefly, ^13^C-labeled cell extracts produced from the continuous cultivation of *M. extorquens* AM1 served as ^13^C-labeled internal standards (^13^C-labeled IS). After fast filtration of *M. trichosporium* OB3b, a fixed amount of ^13^C-labeled IS was added to the petri dish prior to the cell storage. After the metabolites were extracted, they were analyzed by LC-MS/MS and GC-MS as previously described. The calibration curve was developed by adding a fixed amount of ^13^C-labeled IS to different dilutions of primary stock solutions of ^12^C-standard mixtures. Calibration plots were obtained by plotting the ratio of ^12^C-standard to ^13^C-labeled IS versus the ^12^C-standard concentration.

### Dynamic ^13^C-incorporation

For the ^13^CH_4_ labeling experiment, *M. trichosporium* OB3b grown on ^12^C-methane to mid-exponential phase (corresponding to 25% of the added methane consumed) were rapidly transferred to a fresh flask and supplemented with the same percentage of ^13^CH_4_. At the defined time points (0, 1, 2, 5, 10, 20, 40, and 60 min), the cell culture was harvested, and metabolites were analyzed as previously described. The mass isotopomer distributions were corrected for the natural isotope contribution by using a matrix-based method (Yang et al., 2012) and calculated as the relative abundances of the different possible mass isotopomers of a metabolite.

For the ^13^CO_2_ tracing experiment, mid-exponential cultures were spiked with ^13^CO_2_, resulting in 5% ^13^CO_2_ in the headspace. These were also harvested at 0, 0.5, 1, 2, 5, 10, and 20 min, and metabolites were analyzed as previously described.

### Calculation of ^13^C-incorporation rate

Total ^13^C-incorporation of each metabolite was obtained by normalizing to its total carbon number. Relative isotopic abundance (*M*_i_) for a metabolite in which i ^13^C atoms were incorporated was calculated by the Eq. 1:

(1) $$M_{i}(\%)=\frac{m_{i}}{\sum_{j=0}^{n}m_{j}}$$

Where *m*_i_ represented the isotopic abundance for a metabolite in which *i* ^13^C atoms were incorporated and *n* represented the maximum number of ^13^C atoms incorporated.

Total ^13^C-incorporation of a metabolite with N carbon atoms was obtained by normalizing to its total carbon number according to the following Eq. 2:

(2) $$\text{Total}{\;}^{13}\text{C}-\text{incorporation}(\%)=\frac{\sum_{i=1}^{N}\left(i\times M_{i}\right)}{N}$$

^13^C-incorporation rate was calculated from the initial slope of all ^13^C-isotopologues versus time. Comparison of ^13^C-incorporation was obtained by plotting ^13^C-incorporation (%) during a switchover from ^12^CH_4_ to ^13^CH_4_ versus ^13^CO_2_ spiking in a time course. The correlation coefficients (*R^2^*) between ^13^CO_2_ vs. ^13^CH_4_ were calculated using linear regression.

### Cell volume determination

An average OB3b cell volume was determined based on confocal images of OB3b cells stained with the lipid dye FM 1–43 (Invitrogen™). After growing colonies on agar plates, OB3b cells were re-suspended in NMS1 media as described and stained for 1 h at 20°C with FM 1–43 (5 μg/mL). The cells were then washed with NMS1 media, seeded on a poly lysine coated slide, and imaged on a Zeiss Axiovert 200 M microscope with an LSM 510 META confocal attachment with a 100× oil immersion objective (NA = 1.30). Image spacing in the *Z*-axis was 0.5 μm. Complete images of non-mobile cells were analyzed using ImageJ with the Volumest plug-in to determine the average volume (3.79 fL/cell; *n* = 60). The concentration of cells per mL was estimated using a CyFlow^®^ (Partec) flow cytometer with true volumetric absolute counting (TVAC). An average *M. trichosporium* OB3b cell number per 1 mL per OD = 1 is 8.6 × 10^7^ ± 2.6 × 10^6^.

## Conflict of Interest Statement

The authors declare that the research was conducted in the absence of any commercial or financial relationships that could be construed as a potential conflict of interest.

## Appendix

**Table A1:** **Macromolecule and amino acid composition of cells grown on methane**.

Compound		% Cell dry weight	Source of data
C2	PHB	2.5 ± 2	Weaver et al. (1975), Doronina et al. (2008)
	Lipids (FA/PL)	9.2	Weaver et al. (1975)
	Glycine	2.9	This study
C3	DNA/RNA	23	This study
	Serine	2.6	This study
	Cysteine	0.4	This study
	Tryptophan	1.2	This study
	Tyrosine	2	This study
	Phenylalanine	2.9	This study
	Alanine	4.7	This study
	Leucine	5.4	This study
	Valine	3.9	This study
	Cell wall (LPS/PG)	7	Neidhardt et al. (1996)*
C4	Aspartate	3.2	This study
	Asparagine	1.6	This study
	Lysine	2.5	This study
	Methionine	1.3	This study
	Threonine	2.9	This study
	Isoleucine	2.9	This study
C5	Proline	2.5	This study
	Glutamate	3.7	This study
	Glutamine	1.9	This study
	Arginine	5.2	This study
	Histidine	1.4	This study
	Small metabolites, cofactors, ions	3.5	Neidhardt et al. (1996)*
	Total	97.6	

*FA, fatty acids; PL, phospholipids; LPS, lipopolysaccharide; PG, peptidoglycan; PHB, polyhydroxybutyrate*.

**Data for *E. coli*, from Neidhardt et al. (1996)*.
